# Real‐world evaluation of capillary microsampling for drug monitoring of anti‐seizure medications

**DOI:** 10.1002/epi.70439

**Published:** 2026-09-01

**Authors:** Chiara Cancellerini, Alice Caravelli, Martina Soldà, Erika Esposito, Luca Vignatelli, Barbara Mostacci, Jessica Fiori, Francesca Bisulli, Laura Licchetta

**Affiliations:** ^1^ Department of Biomedical and Neuromotor Sciences University of Bologna Bologna Italy; ^2^ IRCCS, Istituto delle Scienze Neurologiche di Bologna, Full Member of the European Reference Network for Rare and Complex Epilepsies (EpiCARE) Bologna Italy; ^3^ Department of Chemistry “G. Ciamician” University of Bologna Bologna Italy

**Keywords:** Anti‐seizure medications (ASMs), epilepsy, quantitative dried blood spot (qDBS), therapeutic drug monitoring (TDM), ultra‐high performance liquid chromatography‐tandem mass spectrometry (UHPLC–MS/MS), volumetric absorptive microsampling (VAMS)

## Abstract

**Objective:**

Capillary microsampling offers a minimally invasive alternative to venipuncture for therapeutic drug monitoring (TDM) of anti‐seizure medications (ASMs). We evaluated the feasibility and reliability of volumetric absorptive microsampling (VAMS) and quantitative dried blood spot (qDBS) devices for ambulatory self‐collection and at‐home use in persons with epilepsy (PwE).

**Methods:**

PwE attending the Epilepsy Centre of the IRCCS‐Istituto delle Scienze Neurologiche di Bologna (Italy) were enrolled between October 2023 and October 2024. Participants performed supervised self‐collection using VAMS and qDBS in ambulatory setting and at‐home self‐collection with VAMS devices. Sample quality, delivery success, and patient‐reported outcomes (ease, pain, and clarity of instructions) were recorded. Carbamazepine, lacosamide, lamotrigine, and levetiracetam were quantified using a validated ultra‐high performance liquid chromatography‐tandem mass spectrometry (UHPLC–MS/MS) method. Reliability was assessed by comparing at‐home VAMS with ambulatory VAMS and cross‐validating qDBS with VAMS and venous plasma samples using Bland–Altman analysis and linear regression.

**Results:**

A total of 105 PwE (66% female, mean age 41 years) performed at‐home and ambulatory self‐collection using VAMS and qDBS devices. Most at‐home VAMS samples (88%) were received by the laboratory within 7 days, and 86.8% met high‐quality criteria. For lacosamide, lamotrigine, and levetiracetam, we found strong correlations between at‐home and ambulatory VAMS (Pearson's *r* ranging from .88 to .98) and low mean bias (<1.0 μg/mL). Conversely, carbamazepine showed lower reliability (*r* = .31; *p* = .49; bias = 2.05 μg/mL). Cross‐validation of qDBS vs plasma confirmed good agreement (slope = .92, .63–1.21) with no significant systematic bias. Patient feedback indicated that self‐collection was easy and minimally painful, although age>60 years impacted sampling quality, particularly for qDBS (*p* = .04).

**Significance:**

VAMS‐based self‐collection is feasible and reliable for at‐home TDM, provided that high‐quality sampling is ensured. qDBS represents a reliable alternative for ambulatory monitoring. Future work should focus on optimizing sampling procedures for older adults and improving logistics for home‐based microsampling.

**Key points**

- Microsampling devices (volumetric absorptive microsampling [VAMS] and quantitative dried blood spot [qDBS]) are reliable tools for therapeutic drug monitoring of anti‐seizure medications (ASMs).
- High‐quality at‐home VAMS samples showed good agreement compared with the VAMS ambulatory for most ASMs.
- Patients reported VAMS self‐collection as feasible, easy, and well‐tolerated.
- Future work should optimize older adult sampling and improve home‐based logistics.

## 1 INTRODUCTION

Since 2015, microsampling devices have emerged as an innovative method for blood collection and are particularly suitable for therapeutic drug monitoring (TDM).^1^, ^2^, ^3^, ^4^, ^5^ These devices, based on fingerprick sampling, allow the collection of minimal sample volumes (<100 μL) with a minimally invasive technique that is considerably less painful than conventional venipuncture.^6^, ^7^, ^8^ A key benefit of microsampling is the potential for ambient temperature storage, which eliminates the requirement for a refrigerated cold chain and maintains analyte stability for several days. This logistical flexibility enables patients to perform at‐home self‐collection and ship samples to a laboratory via standard mail, facilitating remote clinical monitoring and improving patient compliance.^3^, ^9^, ^10^, ^11^ Microsampling for TDM is especially relevant for persons with epilepsy (PwE), where reaching and maintaining individual therapeutic anti‐seizure medication (ASM) concentration levels is fundamental for seizure control and minimizing adverse effects.^1^, ^12^, ^13^ Volumetric absorptive microsampling (VAMS, Mitra) has recently gained attention due to its ability to overcome the hematocrit effect, and its reported strong correlation with plasma concentrations (reference‐standard) for several ASMs, highlighting the analytical performance of VAMS.^4^, ^6^, ^14^, ^15^ However, although studies have confirmed the analytical performance of VAMS, questions remain regarding its real‐world feasibility. Specifically, the reported 7‐day stability window for some ASMs, which may not always be sufficient to accommodate sample shipping and laboratory workflow, preventing the feasibility of at‐home use by PwE.^14^, ^16^, ^17^, ^18^

Furthermore, a newer device based on quantitative dried blood spot (qDBS‐ Capitainer) allows longer sample storage, with improved stability of up to 30 days.^14^, ^19^ Despite the potential of these microsampling methods, there is currently a lack of robust reliability data and cross‐validation against established microsampling methods and venous plasma in the clinical setting.

To address these gaps, we conducted a comprehensive evaluation of capillary microsampling in a cohort of PwE, structuring the study around two primary aims: (1) to evaluate the real‐life feasibility and reliability of self‐collected VAMS samples at home, and (2) to determine the reliability of the qDBS device for monitoring the most commonly prescribed ASMs, compared to the reference‐standard.

## 2 MATERIALS AND METHODS

### 2.1 Population, setting, and procedures

The study protocol was approved by the Independent Ethics Committee of Area Vasta Emilia Centro (approval number 792‐2022‐DISP‐AUSLBO) on January, 18 2023. Adult PwE (≥18 years of age) attending the Epilepsy Centre of the IRCCS Institute of Neurological Sciences of Bologna were considered eligible for the study. All consenting patients treated with carbamazepine (CBZ), lacosamide (LCM), lamotrigine (LTG), and levetiracetam (LEV) as monotherapy or in combination and who were able to independently perform a fingerprick were included in the study. These ASMs were selected because they are among the most prescribed in adult PwE and are monitored routinely by therapeutic drug monitoring, as shown in our previous study.^16^

On the day of enrollment, each participant underwent venous blood sampling in an ambulatory setting before their scheduled ASMs morning dose (see Section 2.3) and performed self‐collected capillary blood sampling using both VAMS and qDBS devices after a healthcare staff training and under their supervision. For the at‐home phase, participants were provided with a sampling kit containing two VAMS devices, illustrated user‐friendly instructions for sample collection, detailed shipment guidelines, and an ad hoc survey based on a 5‐point Likert scale (ranging from 1 = “easy, painless, user‐friendly, or clear,” to 5 = “poor, difficult, painful, not user‐friendly, or unclear”).^9^, ^20^ The survey is provided in supplementary material (Table S1). At‐home VAMS samples were collected under conditions designed to ensure consistency with those of the ambulatory setting, ensuring consistency between the two sampling environments. VAMS samples were shipped to the laboratory through a delivery company or standard mail service, with procedures in place to ensure sample integrity during transport.

The real‐life feasibility of VAMS (Aim 1) was evaluated through patient‐reported outcomes, including ease of sampling, pain perception, operational difficulties encountered during the process, clarity of instructional materials, and mailing procedures. Additional indicators included the proportion of successfully delivered samples, the time elapsed between sample collection and arrival at the laboratory, compliance with the 7‐day stability window, and overall sample integrity upon visual inspection. Reliability of VAMS samples collected at‐home with those obtained in an ambulatory setting was assessed by comparing their ASM concentrations.

Finally, we assess the reliability of the fingerprick device qDBS for ASMs monitoring (Aim 2). The performance of this device was evaluated by a cross‐validation of ASM concentrations obtained by the qDBS device against both venous blood samples and VAMS samples collected in the ambulatory setting.

### 2.2 Chemicals, reagents, solutions, and devices

Standard solutions of CBZ, LTG, and LEV at 1.0 mg/mL in methanol (MeOH) and LCM at 1.0 mg/mL in acetonitrile (ACN) were sourced from Merck Life Science (certified reference material – Cerilliant). As internal standard (IS), CBZ D_10_ solution (100 μg/mL in MeOH), LCM ^13^C‐D_3_ solution (1.0 mg/mL in ACN), LTG ^13^C, ^15^N_4_ solution (500 μg/mL in MeOH), and LEV D_6_ solution (1.0 mg/mL in MeOH), with a purity of ≥99% were provided by Sigma‐Aldrich (St. Louis, Missouri, USA). ACN was provided by VWR Chemicals (Radnor, PA, USA), liquid chromatography‐tandem mass spectrometry (LC–MS/MS) gradient‐grade MeOH provided by Merck Millipore (Darmstadt, Germany), ethanol (EtOH) provided by PanReac AppliChem ‐ ITW Reagents, and formic acid (FA) provided by Carlo Erba Reagents S.r.l. (Milan, Italy). Ultrapure water was obtained from a MilliQ integral apparatus (Merck Millipore), guaranteeing the elimination of impurities that could potentially impact experimental outcomes. Regenerated cellulose membrane .22 μm, 4 mm syringe filters were purchased from Phenomenex (Torrance, CA, USA). The Chromsystems kit (Gräfelfing, Germany) was used for the method cross‐validation.^21^

The VAMS (or volumetric absorptive microsampling) devices, commercially called Mitra, were purchased from Neoteryx (Torrance, CA, USA), whereas qDBS (or quantitative dried blood spot) devices were provided by Capitainer (Stockholm, Sweden), partially free of charge.

The VAMS and qDBS devices used in this study are volumetric, collecting a fixed 10 μL of blood regardless of the subject's hematocrit level. This design choice inherently minimizes the “hematocrit effect,” which is a well‐known limitation of traditional, non‐volumetric dried blood spots.^22^, ^23^, ^24^, ^25^, ^26^

### 2.3 PwE venous blood collection and plasma preparation

Venous blood samples were collected using BD Vacutainer lithium heparin tubes (6 mL capacity), and centrifuged at 3000 rpm for 10 min at 4°C. Plasma samples were stored at −20°C until analysis. Levels of ASMs in plasma were measured using a commercially available kit, following the manufacturer's instructions, and then compared with those obtained from qDBS samples.

### 2.4 Device collection and preparation

VAMS and qDBS samples were collected by PwE under supervised conditions in an ambulatory setting. In addition, VAMS samples were self‐collected at‐home following the user‐friendly instructions provided. Upon arrival at the laboratory, both VAMS and qDBS devices were visually inspected to assess sampling quality. Each sample was evaluated using an aliquot quality score ranging from 0 to 5. For VAMS devices, scores of 0–1 corresponded to samples with less than 30% of the tip volume filled; scores of 2–4 indicated partial filling between 30% and 70% of the nominal volume; and a score of 5 indicated adequate filling exceeding 70% of the nominal volume. For both devices, scores of 0–1 were considered indicative of poor sampling quality, scores of 2–4 reflected partially filled or underfilled samples, and a score of 5 indicated optimal sampling.

Following evaluation, samples were air‐dried for 24 h at room temperature (21°C–25°C) inside a zip‐closure plastic bag with silica gel to ensure proper storage.

VAMS extraction followed previously established methods for ASM microsampling^16^ and for qDBS devices; the pre‐perforated paper discs containing the collected samples were carefully separated using tweezers and individually placed into 2 mL Eppendorf tubes for subsequent analysis. The ASM extraction protocol for the qDBS device was optimized based on our recent publication.^14^ A 0–5 aliquot rating scale was used as a standardized measure of sampling quality for both VAMS and qDBS before ASM extraction, to ensure consistency in assessing both at‐home and ambulatory collections.

### 2.5 Calibration curves and quality control

To ensure accuracy and consistency in concentration levels, reference stock solutions of each ASM were diluted with organic solvent to generate a series of working solution levels. All stock and working solutions were stored at −20°C and used for calibration, providing a comprehensive range for both qDBS and VAMS calibration and quality control (QC) purposes. The final concentration levels prepared in whole blood for each ASM were as follows: for CBZ: 2, 5, 10, 12, and 15 μg/mL; for LCM: 1, 3, 5, 8, and 10 μg/mL; for LTG: 2, 5, 10, 15, and 20 μg/mL; and for LEV: 5, 20, 30, 40, and 50 μg/mL. IS solutions were prepared by diluting the primary standard solution in ACN to achieve the following final IS mixture concentrations: 10 μg/mL for CBZ D_10_, 5 μg/mL for LCM ^13^C‐D_3_, 10 μg/mL for LTG ^13^C, ^15^N4, and 50 μg/mL for LEV D_6_.

Calibration curves and QC samples were prepared for each analytical session. Plasma calibration utilized Chromsystems' multilevel calibration standards, QCs, and IS. Whole blood from healthy volunteers was used to prepare the five calibration points. Spiked blood samples were prepared using these solutions to produce QC samples and calibration standards. For VAMS devices, each tip was touched briefly to the blood surface for 2 s to allow absorption. For qDBS devices, 25 μL of blood was carefully pipetted onto the paper disc to obtain a 10 μL aliquot. Devices were then dried in sealed bags with silica gel packets at room temperature for at least 24 h.

Representative chromatograms showing the separation of the four ASMs for both qDBS and VAMS devices are provided in the Supplementary Material (Figure S1).

### 2.6 UHPLC–MS/MS conditions and method validation

Chromatographic separation and MS quantification were performed according to our previously optimized and validated protocols for ASMs.^14^, ^16^ Briefly, analysis was conducted on a Shimadzu UHPLC system coupled to a Qtrap 4500 Sciex mass spectrometer using a Kinetex C18 column (2.6 μm, 30 × 4.6 mm).

A gradient elution method was employed with a mobile phase consisting of water containing .1% formic acid (FA) (mobile phase A) and acetonitrile containing .1% FA (mobile phase B). The flow rate was set at .6 mL/min, with the column temperature maintained at 30°C and a sample injection volume of 20 μL. For quantification, analysis was performed in both electrospray ionization (ESI)‐positive and ESI‐negative modes using multiple reaction monitoring (MRM) transitions (*m*/*z* = precursor → product ion) specific to each compound.^16^ Nitrogen was used as both source and collision gas. The source temperature was set at 500°C, with an ion spray voltage of 1100 V. The declustering potential (DP), collision energy (CE), entrance potential (EP = 10 V), and collision cell exit potential (CXP = 10 V) were optimized individually for each analyte.

To ensure the analytical robustness of the results, the methods were previously validated following European Medicines Agency (EMA) guidelines for both VAMS^16^ and qDBS.^14^ The validation parameters confirmed high performance for all tested ASMs, specifically CBZ, LCM, LTG, and LEV. Linearity and sensitivity were demonstrated by correlation coefficients (*r* ^2^) of .997 for all compounds, with lower limits of quantitation (LLOQs) established at .5 μg/mL for CBZ and LCM, 1.0 μg/mL for LTG, and 2.0 μg/mL for LEV, effectively covering their respective therapeutic ranges. Intra‐ and inter‐day precision coefficient of variation (CV%) and accuracy relative error (RE%) were consistently within the acceptable limits of ±15% and within ±20% at the LLOQ level.

The extraction efficiency and matrix influence were also rigorously assessed; for VAMS, mean extraction recoveries were >88%, with matrix effects ranging between 92% and 106%, whereas for qDBS, the optimization via Design of Experiments yielded recoveries >85% for all analytes with negligible matrix interference (CV <5%). Furthermore, stability testing confirmed that all analytes remained stable on both devices for at least 7 days at room temperature, supporting the feasibility of home‐sampling and postal shipment. Representative MRM transitions and detailed validation datasets are consistent with those reported in our previous works.^14^, ^16^

### 2.7 Statistical analysis

Agreement between microsampling devices and plasma measurements was assessed using Bland–Altman plots and linear regression, as described previously.^14^, ^16^, ^27^ To evaluate method concordance, we calculated the mean bias with 95% limits of agreement (LoAs), the Pearson's correlation coefficient (*r*), and the coefficient of determination (*R* ^2^).

Proportional bias was assessed by verifying if the 95% confidence interval (CI) of the regression slope included unity (1.0), while systematic bias was evaluated through the 95% CI of the intercept (including zero) and the mean bias from Bland–Altman analysis. Statistical significance for all analyses was set at *p* < .05.

To support the cross‐validation between dried blood and plasma, the blood‐to‐plasma ratio (R‐value) was experimentally determined for each ASM, accounting for the specific partitioning of each drug between erythrocytes and plasma.^28^, ^29^

Analyses were performed using MedCalc Statistical Software (MedCalc Software Ltd., Ostend, Belgium).

## 3 RESULTS

### 3.1 Population

A total of 112 patients were initially enrolled in the study. Of these, nine were excluded due to protocol deviations or analytical errors affecting the collected samples. The final study population included 103 patients (66% female, 34% male; mean age 41 years, range 19–87). Regarding ASM treatment, 18.45% of patients were taking CBZ, 16.50% LCM, 44.66% LTG, and 33.01% taking LEV. Most patients (84.47%) were receiving monotherapy, whereas 12.62% were prescribed polytherapy regimens (Table 1).

**TABLE 1:** Demographic characteristics, clinical data, and microsampling quality assessment.

Patient data		ASMs	
Patients	*n* = 103	Monotherapy (%)	84.5%
Mean age (range)	40.8 (19–87)	Polytherapy (%)	12.6%
Female (%)	66%	LTG (%)	44.7%
Male (%)	34%	LEV (%)	33.0%
		CBZ (%)	18.5%
		LCM (%)	16.5%

Microsampling quality score			
Score	VAMS ambulatory (*n* = 103)	VAMS at‐home (*n* = 91)	qDBS (*n* = 103)
Score 5	86 (83.5%)	67 (73.6%)	50 (48.5%)
Score 4	13 (12.6%)	7 (7.7%)	11 (10.7%)
Score 3	4 (3.9%)	6 (6.6%)	13 (12.6%)
Score 0–2	0 (.0%)	11 (12.1%)	29 (28.1%)

Sub‐analysis of optimal quality (score 5) by age			
	VAMS ambulatory (% of score 5)	VAMS at‐home (% of score 5)	qDBS (% of score 5)
Age 18–30 years	92.3%	55.6%	62.9%
Age 31–50 years	76.5%	64.0%	45.1%
Age 51–70 years	84.8%	70.0%	40.0%
Age>70 years	100.0%	100.0%	33.3%
Total (all ages)	83.5% (86/103)	73.6% (67/91)	48.5% (50/103)

*Note*: Data for the total cohort (*n* = 103). Quality scores compare ambulatory sampling (*n* = 103) vs at‐home self‐collection (*n* = 91). Score 5: optimal; score 0–2: poor quality.

Abbreviations: ASM, antiseizure medication; CBZ, carbamazepine; CV, coefficient of variation; LCM, lacosamide; LEV, levetiracetam; LLOQ, lower limit of quantitation; LTG, lamotrigine; PwE, persons with epilepsy; qDBS, quantitative dried blood spot; TDM, therapeutic drug monitoring; VAMS, volumetric absorptive microsampling.

### 3.2 VAMS real‐life feasibility and reliability: Comparison between VAMS in an ambulatory setting vs VAMS at home (Aim 1)

Of the 103 enrolled patients, 91 VAMS at‐home samples (88.35%) were received successfully at the laboratory for analysis. The interval between the self‐collection of microsamples and their arrival at the laboratory was assessed as ideal if within 7 days after fingerprick sampling. Of the successfully received samples, 86.81% (*n* = 79) arrived within this target period, whereas 8.79% (*n* = 8) arrived after 7 days. In four cases (4.40%), the shipment date was not specified. In addition, 12 devices (11.65%) did not reach the laboratory (see Table S2). Regarding sample quality, VAMS devices collected in an ambulatory setting displayed the following distribution: no samples received a score of 0–1–2, less than 5% were rated as 3, 10.7% of samples were assigned a score of 4, whereas the majority (83.50%) received the highest score of 5 (see Section 2.4).

Among the VAMS devices collected at home, two samples received a score of 0 and 1, respectively. Less than 10% received a score of 2 (9.89%), 3 (6.59%), and 4 (7.69%). Most at‐home samples (73.63%) obtained a maximum score of 5. All data are summarized in Table 1 and in Figure S2.

The ad hoc survey rating the feasibility of at‐home sampling was filled out by 85 of the 91 PwE who sent the sample to the laboratory. Seventy‐seven PwE (90.59%) found the procedure easy (rating 1), whereas a few experienced moderate to great difficulty (5 patients rated 2, 2 rated 3, and 1 rated 5). All PwE but one reported no pain to moderate pain perception (rating 1–3). More than 86% found the instruction notes provided ease of use, and more than 75% considered the shipping instructions clear. Ninety percent of patients watched the video tutorial.

Delivery company and standard mail service were used in a nearly equal manner, whereas four patients delivered their samples, skipping the shipping process (Table 2).

**TABLE 2:** Real‐life feasibility study: Patient‐reported outcomes and feasibility of at‐home VAMS collection.

Survey question (*n* = 85)			
	Positive rating (1–2)	Neutral/negative (3–5)	Missing/NA
Sample collection procedure difficulty	82 (96.5%)	3 (3.5%)	0 (.0%)
Pain during procedure	82 (96.5%)	3 (3.5%)	0 (.0%)
Ease of operating instructions	76 (89.4%)	7 (8.2%)	2 (2.4%)
Clarity of shipping instructions	72 (84.7%)	10 (11.8%)	3 (3.5%)
Watched video tutorial	Yes: 77 (90.6%)	No: 3 (3.5%)	Missing: 5 (5.9%)
Shipment method	Delivery company: 43 (50.6%)	Mail: 38 (44.7%)	Personal: 4 (4.7%)

*Note*: Summary of survey responses regarding the feasibility of the VAMS self‐collection procedure. Procedure difficulty and pain were rated on a 5‐point Likert scale from 1 (very easy/no pain) to 5 (very difficult/severe pain). Instruction clarity was rated from 1 (very clear) to 5 (not clear). For analysis, ratings were condensed into positive (scores 1–2) and neutral/negative (scores 3–5) categories. Percentages are calculated based on the number of respondents (*n* = 85). Missing/NA indicates questions left unanswered. The table also summarizes compliance with the video tutorial and the distribution of shipment methods used by participants.

Abbreviations: NA, not available; VAMS, volumetric absorptive microsampling.

To assess the reliability of VAMS at home compared with VAMS in an ambulatory setting (Aim 1), we initially considered devices with an aliquot quality score≥3 (see Table S3 and Figure S3). Across these samples, the degree of agreement varied notably. LCM and LTG showed consistent correlation, even with quality scores of 3 or 4, whereas CBZ and LEV exhibited lower coefficients of determination (*R* ^2^) (see Section 2.7). Overall, these results indicate that including aliquots with suboptimal quality (scores “3–4”) introduces considerable variability. Statistically significant agreement between sampling settings was observed primarily when analyses were restricted to aliquots with an optimal quality score of “5.” Bland–Altman plots (a) and linear regression (b) results for these high‐quality samples are presented in Table 3 and Figure 1.

**TABLE 3:** Reliability of at‐home VAMS collection: Bland–Altman and linear regression analyses.

	Bland–Altman		Pearson's r	Linear regression						
	Bias	95% CI			*R* ^2^	Intercept	95% CI	Slope	95% CI	*p‐*value
CBZ (*n* = 7)	2.05	−4.03 to 8.12	.32	*y* = 6.3534 + .4910 *x*	.10	6353	−8.535 to 21.242	.491	−1.229 to 2.212	*p* = .4962
LCM (*n* = 10)	.6	−.90 to 2.11	.92	*y* = .9548 + .9092 *x*	.85	.955	−.388 to 2.297	.909	.597 to 1.222	*p* = .0002
LTG (*n* = 29)	0.97	−3.95 to 3.05	.98	*y* = 1.0307 + .8321 *x*	.97	1030	.3844 to 1.6770	.8321	.7785 to .8858	*p* = .0461
LEV (*n* = 20)	−0.56	−7.17 to 6.04	.88	*y* = 2.3375 + .7000 *x*	.78	2338	.131 to 4.544	.7	.513 to .887	*p* = .0156

*Note*: Reliability assessment comparing at‐home self‐collection vs supervised ambulatory collection for VAMS samples with optimal quality (score 5). Bland–Altman analysis reports the mean bias and 95% limits of agreement. Linear regression analysis includes Pearson's r, coefficient of determination (*R* ^2^), intercept, slope, and respective 95% confidence intervals (CIs). The *p*‐value indicates the significance of the correlation.

Abbreviations: CBZ, carbamazepine; LCM, lacosamide; LEV, levetiracetam; LTG, lamotrigine; VAMS, volumetric absorptive microsampling.

**FIGURE 1:**
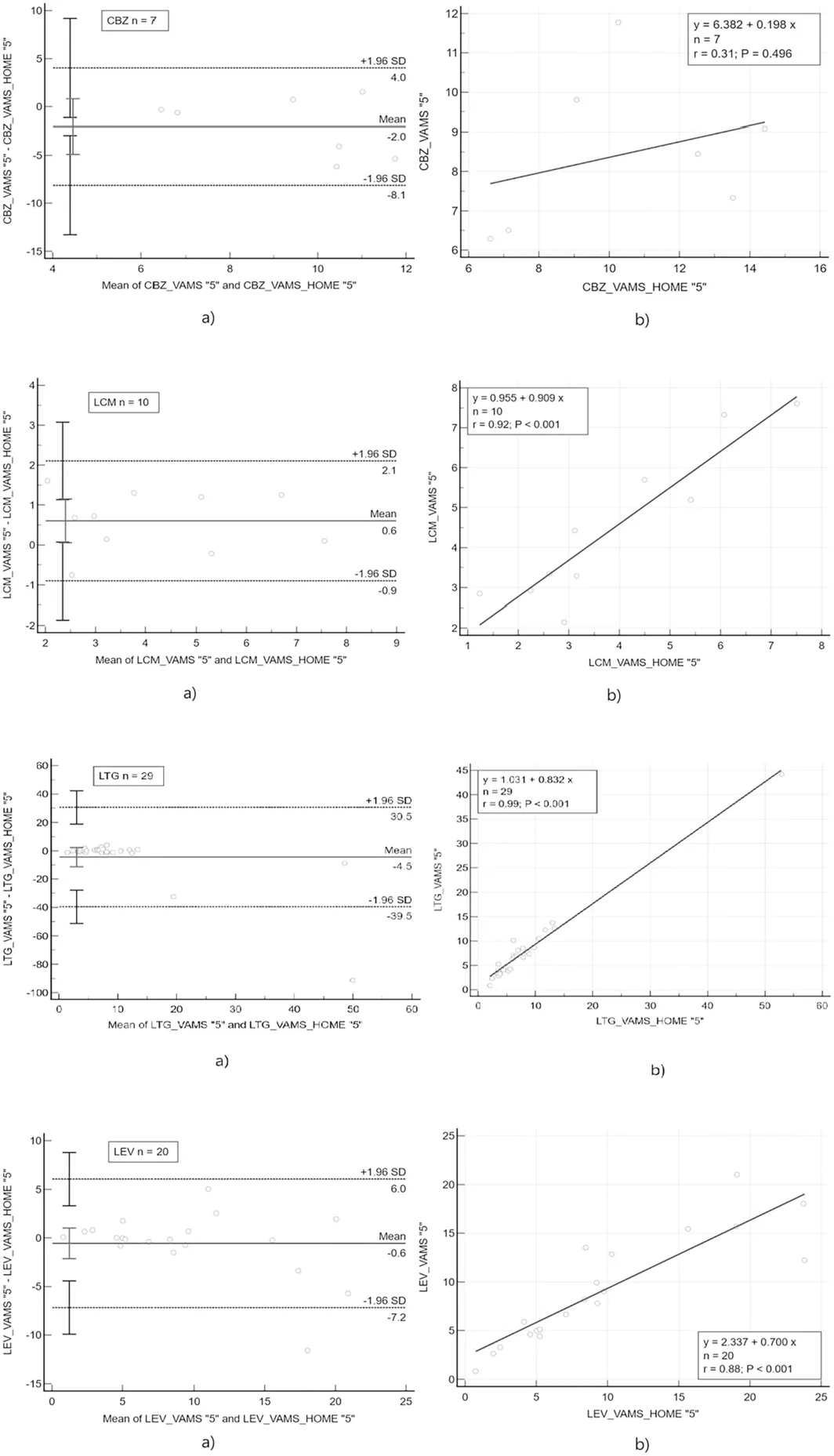
Bland–Altman analysis and linear regression for Aim 1. (A) Bland–Altman plots and (B) linear regression analysis comparing ASM concentrations in at‐home vs ambulatory VAMS samples. The analysis includes the four ASMs studied: carbamazepine (CBZ, *n* = 19), lacosamide (LCM, *n* = 17), lamotrigine (LTG, *n* = 46), and levetiracetam (LEV, *n* = 34). Only samples with optimal quality (score 5) were included in the comparison. In A, the solid line represents the mean difference (bias), whereas the dashed lines indicate the upper and lower limits of agreement (LoAs) with 95% confidence intervals. In B, the X‐axis shows values measured with at‐home VAMS, and the Y‐axis shows values from ambulatory VAMS; the solid line represents the regression, and the dashed lines indicate the 95% confidence intervals. ASM, antiseizure medication; CBZ, carbamazepine; LCM, lacosamide; LEV, levetiracetam; LoA, limits of agreement; LTG, lamotrigine; VAMS, volumetric absorptive microsampling.

Considering only aliquots with a quality score of 5, agreement between at‐home and ambulatory sampling improved for most analytes. Under these conditions, LCM exhibited low mean bias and a regression slope including unity (slope = .909, 95% CI = .597–1.222). LTG showed the highest degree of concordance, with a coefficient of determination (*R* ^2^ = .97) and a narrow CI for the slope. In contrast, LEV, despite maintaining a strong correlation, showed a statistically significant proportional bias (slope = .700, 95% CI = .513–.887). Furthermore, CBZ reliability could not be definitively established due to the limited sample size (*n* = 7), which resulted in an *R* ^2^ = .10 and a *p*‐value of .4962.

Regarding sampling quality, VAMS devices collected in an ambulatory setting showed no poor‐quality samples (scores 0–2), with 83.5% achieving the optimal score of 5. Comparison of sampling quality across settings showed that although no ambulatory samples were rated as poor (score 0–2), 12.1% of at‐home samples fell into this category; nonetheless, a high frequency of optimal quality (score 5) was maintained even in the self‐collection context (73.6% at home vs 83.5% ambulatory).

The relationship between patient age and microsampling performance was also examined. VAMS quality scores followed a non‐linear pattern, with higher proportions of valid samples in younger (18–30 years) and older (>70 years) participants compared to intermediate age groups. In contrast, qDBS quality progressively declined with age and remained low in individuals over 70 years, despite the optimal performance observed with VAMS in the same age group.

### 3.3 Reliability of qDBS (Aim 2)

Ambulatory qDBS devices collected from the 103 subjects in an ambulatory setting demonstrated a variable range of quality scores (Table 1). Almost half of the samples (48.5%) were scored 5, indicating optimal sample collection; 10% of the devices scored a 4, whereas 12% scored a 3. Notably, 18% recorded a score of 0. A cross‐validation analysis compared ASM concentrations from qDBS with both plasma and ambulatory VAMS collected at the same time in an ambulatory setting (Figure 2).

**FIGURE 2:**
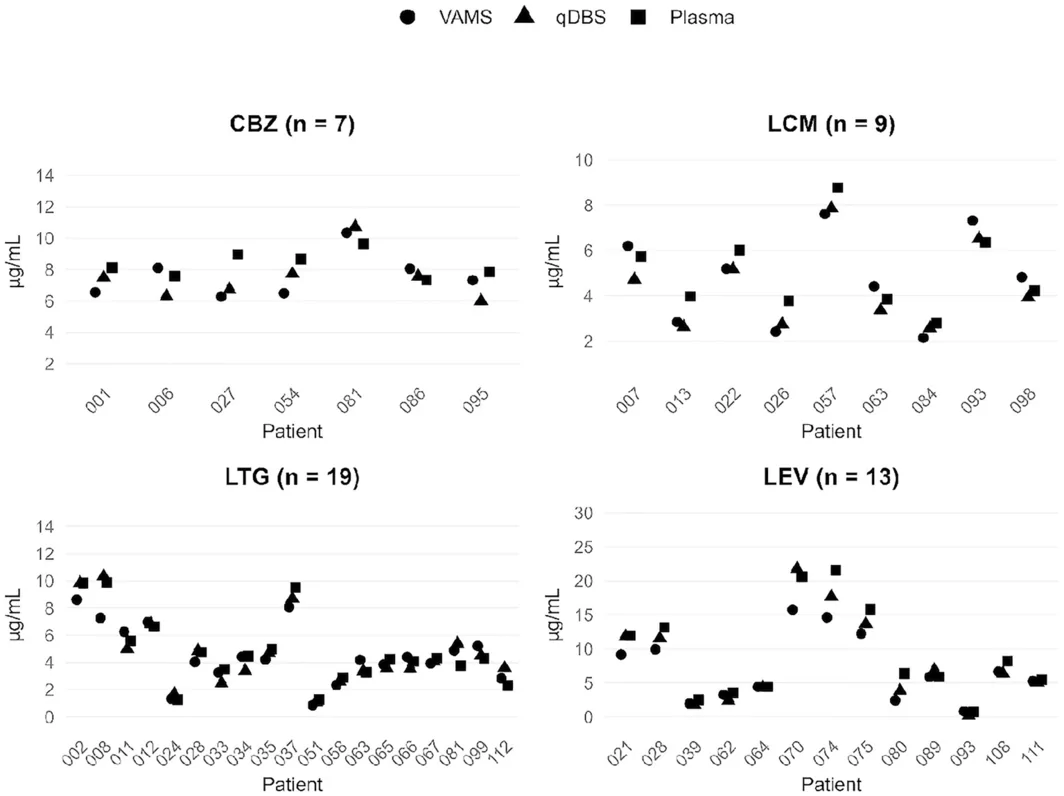
Comparison of ASM concentrations measured in plasma, qDBS, and VAMS samples collected in ambulatory settings. Comparison of ASM concentrations measured in plasma (■), qDBS (▲), and VAMS (●) samples collected in ambulatory settings for individual patients. The four panels represent results for carbamazepine (CBZ), lacosamide (LCM), lamotrigine (LTG), and levetiracetam (LEV).

*R*‐value was experimentally calculated for each ASM by comparing microsampling and corresponding plasma yielding values of .89 ± .18 for CBZ, .89 ± .20 for LCM, .74 ± .21 for LEV and 1.03 ± .34 for LTG for VAMS, and .79 ± .28 for CBZ, .78 ± .27 for LCM, .77 ± .26 for LEV, and .92 ± .29 for LTG for qDBS.^29^

Linear regression and Bland–Altman analysis showed that no statistically significant systematic bias was present in the comparison between qDBS and plasma or between qDBS and VAMS, as the 95% CI of the mean bias consistently included zero (−2.35 to 2.18 for CBZ and −2.59 to 4.90 for LEV; Table S4). qDBS vs plasma data showed strong correlation coefficients (*R* ^2^ ranging from .50 to .97), in line with our previous findings.^14^ Agreement between qDBS and VAMS was further confirmed when comparing only optimal quality samples (score 5), with regression slopes showing no significant proportional bias for LCM (slope 95% CI = .585–1.138) (Figure S4 and Table S4).

## 4 DISCUSSION

Our study demonstrates that at‐home VAMS self‐collection is a feasible and reliable strategy for TDM in PwE, provided that sample quality is strictly controlled. The high return rate (88%) and positive patient feedback align with previous findings, suggesting that microsampling is well‐tolerated and preferred over venipuncture.

Regarding feasibility and logistics, a critical finding was to perform the shipment and analysis in 7‐day stability window. Although 86.8% of samples arrived on time, the failure of nearly 12% of samples to reach the lab highlights a significant logistical challenge. Unlike clinical settings where transport is standardized, at‐home TDM relies on postal services, which may introduce delays, potentially affecting analyte stability, as reported previously for certain ASMs.^30^

The role of sample quality is paramount, as our results emphasize that analytical reliability is strictly dependent on sampling quality. The significant improvement in correlation when restricting analysis to “score 5” samples suggests that visually suboptimal samples (scores 3–4) should be interpreted with caution or rejected. This is particularly relevant for LTG, which showed excellent concordance (*R* ^2^ = .97) only under optimal conditions. The poor performance of CBZ in our cohort likely reflects the limited sample size rather than an inherent failure of the device, although further studies are needed to confirm this.

Of interest, although VAMS performance remained stable across age groups, qDBS quality declined significantly in patients age 70 or older. This suggests that the technical requirements for correct qDBS collection (e.g., maintaining a specific angle or pressure) may be more challenging for elderly patients, whereas the “wicking” action of VAMS is more intuitive.

The cross‐validation of qDBS against plasma and VAMS confirms its reliability in an ambulatory setting. The lack of systematic bias and the experimental R‐value close to 1 for the tested ASMs support the use of qDBS as a valid alternative to traditional sampling, offering a wider stability window (up to 30 days), which could mitigate the logistical issues observed with VAMS.

Overall, the data indicate that concentrations obtained from qDBS closely align with those from plasma samples and VAMS, although some variability is observed among individual patients. qDBS concentrations may slightly under‐ or overestimate plasma values in certain cases, but no systematic bias was found. This is consistent with the good correlation observed between qDBS and plasma measurements, which has been confirmed in previous studies.^14^

When considering the integration of microsampling into routine TDM, the choice between VAMS and qDBS depends on the clinical setting.^4^, ^31^ Our results suggest that VAMS is highly intuitive for patients of all ages, as shown by the stable quality scores even in the elderly. Its “wicking” mechanism minimizes user error in volume collection.

Although this study focused on four commonly prescribed ASMs (CBZ, LCM, LTG, and LEV), the validated ultra‐high performance liquid chromatography‐tandem mass spectrometry (UHPLC–MS/MS) platform and the microsampling techniques described are potentially applicable to a broader range of ASMs.

However, caution is required for drugs with high protein binding or significant red blood cell partitioning, where the blood‐to‐plasma ratio must be rigorously pre‐determined.

We are aware of the limitations of the study. The suboptimal agreement observed for CBZ (*R* ^2^ = .10) warrants careful consideration. Unlike LTG and LEV, CBZ exhibits a more complex pharmacokinetics profile and a narrower therapeutic window, making its plasma concentrations highly sensitive to the exact timing of sampling relative to the last dose. Although patients were instructed to perform at‐home VAMS collection under trough conditions (immediately before the morning dose), the lack of a verified patient diary to document the exact dosing‐to‐sampling interval represents a limitation of this real‐world study. In non‐supervised settings, minor fluctuations in the timing of self‐collection may introduce significant analytical variability for drugs with shorter half‐lives or rapid absorption phases. Future studies implementing digital diaries or timestamped collection devices may help further clarify the impact of patient compliance on the reliability of at‐home TDM for medications like CBZ.^32^

## 5 CONCLUSIONS

This study evaluated the real‐life feasibility and reliability of two microsampling devices, VAMS and qDBS, for potential implementation in clinical routine, focusing on PwE treated with different ASMs at the Istituto delle Scienze Neurologiche di Bologna (IRCCS‐ISNB) Institute of Neurological Sciences, Bellaria Hospital, Bologna.

Our findings demonstrate that the clinical reliability of capillary microsampling is strictly dependent on rigorous quality assurance. Strong concordance with reference methods was achieved for LCM, LTG, and LEV, but only when analysis was strictly restricted to optimal‐quality aliquots (score 5), highlighting that visual sample inspection must serve as a primary gatekeeper in laboratory workflows. Cross‐validation confirmed that qDBS provides ASM concentrations comparable to VAMS and plasma, with no systematic bias, supporting its use as an alternative capillary blood collection method in ambulatory settings.

VAMS at‐home self‐collection proved largely feasible and well‐accepted: most samples arrived within 7 days, and the majority achieved the highest quality score. Patient feedback highlighted ease of use, minimal pain, and good adherence to instructions, although logistical challenges related to shipping remain a limitation.

However, sample quality and analyte measurements were significantly influenced by the collection environment and patient age. Notably, older adults demonstrated suboptimal performance and lower sampling quality, particularly with the qDBS device, highlighting that future clinical implementation requires optimizing home‐based postal logistics and refining sampling procedures for elderly populations.

In conclusion, VAMS‐based self‐collection is feasible and well tolerated for at‐home TDM, provided that high‐quality sampling and standardized procedures are ensured. Future studies should optimize sampling with qDBS in older adults and refine logistics for home‐based microsampling. Finally, it is warranted to conduct a study on the change in management, comparing the clinician's decision based on the fingerprick test result with that based on the standard plasma test.

## AUTHOR CONTRIBUTIONS

Chiara Cancellerini: Conceptualization, data curation, formal analysis, investigation, methodology, writing – original draft, writing – review & editing. Alice Caravelli: Data curation, formal analysis, investigation, writing – review & editing. Martina Soldà: data curation, investigation. Erika Esposito: data curation, investigation, writing – review & editing. Luca Vignatelli: Conceptualization, methodology, supervision, writing – review & editing. Barbara Mostacci: Supervision, writing – review & editing. Jessica Fiori: Investigation, supervision, writing – review & editing. Francesca Bisulli: Conceptualization, supervision, writing – review & editing. Laura Licchetta: Conceptualization, methodology, investigation, supervision, writing – original draft, writing – review & editing.

## FUNDING INFORMATION

This work was supported by the Fondazione LICE (Italian League Against Epilepsy) [grant to Laura Licchetta].

## CONFLICT OF INTEREST STATEMENT

The authors declare that they have no conflicts of interest related to this manuscript. We confirm that we have read the Journal's position on issues involved in ethical publication and affirm that this report is consistent with those guidelines.

## ETHICS APPROVAL

The study protocol was approved by the Independent Ethics Committee of Area Vasta Emilia Centro (approval number 792‐2022‐DISP‐AUSLBO) on January 18, 2023. All procedures were performed in accordance with the ethical standards of the institutional research committee and with the 1964 Helsinki Declaration and its later amendments. Written informed consent was obtained from all individual participants included in the study.

## Supporting information


**Table S1:** Ad hoc survey for persons with epilepsy (PwE).
**Table S2:** Rate of volumetric absorptive microsampling (VAMS) at‐home sample shipment success, calculated on 103 collected samples, and timing of arrival based on 91 samples received. Percentages are reported as proportion of total collected samples (*) or proportion of samples received (**).
**Table S3:** Reliability analysis for Aim 1: Bland–Altman and linear regression results comparing VAMS samples collected in ambulatory vs at‐home settings with an aliquot quality score≥3. (See Figure S2.)
**Table S4:** Reliability analysis for Aim 2: Bland–Altman and linear regression results comparing qDBS and VAMS samples collected in an ambulatory setting (quality score 5).
**Figure S1:** Representative UHPLC–MS/MS total ion chromatograms (TICs) of calibration standards (Level 3) extracted from (A) VAMS and (B) qDBS devices. The peaks show consistent chromatographic behavior and separation for the four anti‐seizure medications (ASMs) across both sampling platforms. Retention times (RTs) are: LEV (1.88 min), LCM (2.82 min), LTG (2.98 min), and CBZ (3.69 min). Optimal chromatographic separation was achieved for all monitored ASMs and their respective internal standards within a 5.5‐min run.
**Figure S2:** Histogram showing the distribution of VAMS quality percentages for samples collected in ambulatory settings and at home, allowing visual comparison of quality across the two collection contexts.
**Figure S3:** Bland–Altman analysis (A) and linear regression (B) for Aim 1, comparing ambulatory and at‐home VAMS samples with an aliquot quality score≥3, assessing reliability between collection settings.
**Figure S4:** Bland–Altman analysis (A) and linear regression (B) for Aim 2, comparing qDBS and VAMS samples collected in an ambulatory setting, assessing reliability between the two microsampling methods.

## Data Availability

The datasets generated during the current study are available from the corresponding author on reasonable request.

## References

[1] Johannessen Landmark C , Johannessen SI , Patsalos PN . Therapeutic drug monitoring of antiepileptic drugs: current status and future prospects. Expert Opin Drug Metab Toxicol. 2020;16(3):227–238. 10.1080/17425255.2020.1724956 32054370

[2] Patsalos PN , Spencer EP , Berry DJ . Therapeutic drug monitoring of antiepileptic drugs in epilepsy: a 2018 update. Ther Drug Monit. 2018;40(5):526–548. 10.1097/FTD.0000000000000546 29957667

[3] Delahaye L , Veenhof H , Koch BCP , Alffenaar JWC , Linden R , Stove C . Focus series: alternative sampling strategies: alternative sampling devices to collect dried blood microsamples: state‐of‐the‐art. Ther Drug Monit. 2021;43(3):310–321. 10.1097/FTD.0000000000000864 33470777

[4] Idzikowska K , Gątarek P , Kałużna‐Czaplińska J . Modern analytical techniques in epilepsy research. Int J Mol Sci. 2026;27(5):2395. 10.3390/ijms27052395 PMC12986452 41828613

[5] Kok MGM , Fillet M . Volumetric absorptive microsampling: current advances and applications. J Pharm Biomed Anal. 2018;147:288–296. 10.1016/j.jpba.2017.07.029 28803682

[6] D'Urso A , Rudge J , Patsalos PN , de Grazia U . Volumetric absorptive microsampling: a new sampling tool for therapeutic drug monitoring of antiepileptic drugs. Ther Drug Monit. 2019;41(5):681–692. 10.1097/FTD.0000000000000652 31095069

[7] Cafaro A , Conti M , Pigliasco F , Barco S , Bandettini R , Cangemi G . Biological fluid microsampling for therapeutic drug monitoring: a narrative review. Biomedicine. 2023;11(7):1962. 10.3390/biomedicines11071962 PMC10377272 37509602

[8] Velghe S , Stove CP . Volumetric absorptive microsampling as an alternative tool for therapeutic drug monitoring of first‐generation anti‐epileptic drugs. Anal Bioanal Chem. 2018;410(9):2331–2341. 10.1007/s00216-018-0866-4 29362853

[9] Van Uytfanghe K , Heughebaert L , Stove CP . Self‐sampling at home using volumetric absorptive microsampling: coupling analytical evaluation to volunteers' perception in the context of a large scale study. Clin Chem Lab Med. 2020;59(5):e185–e187. 10.1515/cclm-2020-1180 33112777

[10] Zhao J , Setchell KDR , Zhao X , Galandi S , Garr BAN , Gao Z , et al. Use of volumetric absorptive microsampling and parallel reaction monitoring mass spectrometry for tacrolimus blood trough measurements at home in pediatric heart transplant patients. J Mass Spectrom Adv Clin Lab. 2024;31:1–7. 10.1016/j.jmsacl.2023.11.004 PMC10755538 38163003

[11] Licchetta L , Trivisano M , Baldin E , Mohamed S , Raschi E , Mostacci B , et al. TELEmedicine for EPIlepsy Care (TELE‐EPIC): protocol of a randomised, open controlled non‐inferiority clinical trial. BMJ Open. 2021;11(12):1–8. 10.1136/bmjopen-2021-053980 PMC8646970 34862297

[12] Patsalos PN , Berry DJ , Bourgeois BF , Cloyd JC , Glauser TA , Johannessen SI , et al. Antiepileptic drugs‐best practice guidelines for therapeutic drug monitoring: a position paper by the subcommission on therapeutic drug monitoring, ILAE Commission on Therapeutic Strategies. Epilepsia. 2008;49(7):1239–1276. 10.1111/j.1528-1167.2008.01561.x 18397299

[13] Delahaye L , Ogwang R , Hotterbeekx A , Colebunders R , Mandro M , Idro R . Dried blood microsampling‐based therapeutic drug monitoring of antiepileptic drugs in children with nodding syndrome and epilepsy in Uganda and The Democratic Republic of the Congo. Ther Drug Monit. 2020;42(3):481–490. 10.1097/FTD.0000000000000720 31834012

[14] Cancellerini C , Caravelli A , Esposito E , Beatrice Belotti LM , Soldà M , Derus N , et al. Quantitative dried blood spot microsampling for therapeutic drug monitoring of ‐antiseizure medications by design of experiment and UHPLC‐MS/MS. Talanta. 2025;293:128018. 10.1016/j.talanta.2025.128018 40157158

[15] Klotz KA , Schulze‐Bonhage A , Antonio‐Arce VS , Jacobs J . Cannabidiol for treatment of childhood epilepsy—a cross‐sectional survey. Front Neurol. 2018;9:1–7. 10.3389/fneur.2018.00731 PMC6143823 30258395

[16] Cancellerini C , Belotti LMB , Mohamed S , Solda’ M , Esposito E , Bisulli F , et al. Fingerprick volumetric absorptive microsampling for therapeutic drug monitoring of antiseizure medications: reliability and real‐life feasibility in epilepsy patients. J Pharm Biomed Anal. 2024;242:116065. 10.1016/j.jpba.2024.116065 38401349

[17] Mohamed S , Perrone A , Cancellerini C , Esposito E , Caravelli A , Donadio V , et al. Volumetric absorptive microsampling coupled with UHPLC‐MS/MS for the determination of Lyso‐Gb3 as Fabry disease diagnostic biomarker. Microchem J. 2024;200:110494.

[18] Mbughuni MM , Stevens MA , Langman LJ , Kudva YC , Sanchez W , Dean PG , et al. Volumetric microsampling of capillary blood spot vs whole blood sampling for therapeutic drug monitoring of tacrolimus and cyclosporin a: accuracy and patient satisfaction. J Appl Lab Med. 2020;5(3):516–530. 10.1093/jalm/jfaa005 32445361

[19] Deprez S , Van Uytfanghe K , Stove CP . Liquid chromatography‐tandem mass spectrometry for therapeutic drug monitoring of immunosuppressants and creatinine from a single dried blood spot using the Capitainer® qDBS device. Anal Chim Acta. 2023;1242:340797. 10.1016/j.aca.2023.340797 36657891

[20] Protti M , Mandrioli R , Mercolini L . Tutorial: Volumetric absorptive microsampling (VAMS). Anal Chim Acta. 2019;1046:32–47. 10.1016/j.aca.2018.09.004 30482302

[21] International Council for Harmonisation (ICH) . Guideline Q2(R2) on validation of analytical procedures. Amsterdam, The Netherlands: European Medicines Agency; 2023.

[22] Velghe S , Delahaye L , Stove CP . Is the hematocrit still an issue in quantitative dried blood spot analysis? J Pharm Biomed Anal. 2019;163:188–196. 10.1016/j.jpba.2018.10.010 30317075

[23] Velghe S , Stove CP . Evaluation of the Capitainer‐B microfluidic device as a new hematocrit‐independent alternative for dried blood spot collection. Anal Chem. 2018;90(21):12893–12899. 10.1021/acs.analchem.8b03512 30256092

[24] De Kesel PMM , Sadones N , Capiau S , Lambert WE , Stove CP . Hemato‐critical issues in quantitative analysis of dried blood spots: challenges and solutions. Bioanalysis. 2013;5(16):2023–2041. 10.4155/bio.13.156 23937137

[25] Luginbühl M , Gaugler S . Addressing new possibilities and new challenges: automated nondestructive hematocrit normalization for dried blood spots. Ther Drug Monit. 2021;43(1):15–22. 10.1097/FTD.0000000000000887 33973966

[26] Daousani C , Karalis V , Malenović A , Dotsikas Y . Hematocrit effect on dried blood spots in adults: a computational study and theoretical considerations. Scand J Clin Lab Invest. 2019;79(5):325–333. 10.1080/00365513.2019.1622033 31154908

[27] Bland JM , Altman DG . Statistical methods for assessing agreement between two methods of clinical measurement. Lancet. 1986;1(8476):307–310. 2868172

[28] Mamada H , Iwamoto K , Nomura Y , Uesawa Y . Predicting blood‐to‐plasma concentration ratios of drugs from chemical structures and volumes of distribution in humans. Mol Divers. 2021;25(3):1261–1270. 10.1007/s11030-021-10186-7 PMC8342319 33569705

[29] Boffel L , De Baets H , Heughebaert L , Rispens T , Åsberg A , Shipkova M , et al. To convert or not to convert? Official International Association of Therapeutic Drug Monitoring and Clinical Toxicology Guideline: considerations and recommendations for converting capillary blood microsampling concentrations to plasma concentrations. Ther Drug Monit. 2026;48(1):1–26. 10.1097/FTD.0000000000001393 41191790

[30] Gustavsen MT , Midtvedt K , Vethe NT , Robertsen I , Bergan S , Åsberg A . Tacrolimus area under the concentration versus time curve monitoring, using home‐based volumetric absorptive capillary microsampling. Ther Drug Monit. 2020;42(3):407–414. 10.1097/FTD.0000000000000697 31479042

[31] Canisius TPIJM , Soons JWPH , Verschuure P , Wammes‐Van Der Heijden EA , Rouhl RPW , Majoie HJM . Therapeutic drug monitoring of anti‐epileptic drugs—a clinical verification of volumetric absorptive micro sampling. Clin Chem Lab Med. 2020;58(5):828–835. 10.1515/cclm-2019-0784 32045349

[32] Verougstraete N , Lapauw B , Van Aken S , Delanghe J , Stove C , Stove V . Volumetric absorptive microsampling at home as an alternative tool for the monitoring of HbA1c in diabetes patients. Clin Chem Lab Med. 2017;55(3):462–469. 10.1515/cclm-2016-0411 27732552

