# Supplementary material for: Real‐world evaluation of capillary microsampling for drug monitoring of anti‐seizure medications
Source: Epilepsia. 2026 Sep 1;67(11):5703–14. doi: 10.1002/epi.70439 (PMC13768884; doi:10.1002/epi.70439)
Supplement: Supplementary file 1 [file EPI-67-5703-s001.docx]

**Real‐world evaluation of capillary microsampling for drug monitoring of antiseizure medications**

Chiara Cancellerini^1^, Alice Caravelli^2^, Martina Soldà^2^, Erika Esposito^2^, Luca Vignatelli^2^, Barbara Mostacci^2^, Jessica Fiori^2,3^, Francesca Bisulli^1,2^, Laura Licchetta^2^

^1^Department of Biomedical and Neuromotor Sciences, University of Bologna, Bologna, Italy

^2^IRCCS, Istituto delle Scienze Neurologiche di Bologna, Full Member of the European Reference Network for Rare and Complex Epilepsies (EpiCARE), Bologna, Italy

^3^Department of Chemistry “G. Ciamician”, University of Bologna, Italy

Corresponding author:

Francesca Bisulli MD, PhD

Associate Professor of Neurology

Department of Biomedical and NeuroMotor Sciences, Alma Mater Studiorum – University of Bologna

Director of Epilepsy Program, IRCCS Istituto delle Scienze Neurologiche, Azienda USL di Bologna.

Lead of the EpiCARE-ERN full Member Centre of Bologna

Pad G1 - Ospedale Bellaria

Via Altura 3 - 40139 Bologna

e-mail: [francesca.bisulli@unibo.it](mailto:francesca.bisulli@unibo.it)

**SUPPLEMENTARY MATERIAL**

**Table S1:** *Ad hoc* survey for persons with epilepsy (PwE).

| **Assessment Item** | **Question / Prompt** | **Rating Scale / Options** |
| --- | --- | --- |
| Procedure Difficulty | Did you find the sample collection procedure DIFFICULT? | 1 (Easy) to 5 (Difficult) |
| Pain Perception | Did you experience PAIN during the procedure? | 1 (Little) to 5 (A lot) |
| Operating Instructions | Did you find the OPERATING INSTRUCTIONS for the sample collection easy to use? | 1 (Very easy) to 5 (No, not easy at all) |
| Shipping Instructions | Did you find the shipping instructions clear? | 1 (Yes, very clear) to 5 (No, not clear at all) |
| Video Tutorial Use | Did you need to watch the video tutorial? | □ Yes / □ No |
| Timing | Home sample collection date and time | [Open entry] |

**Table S2:** Rate of volumetric absorptive microsampling (VAMS) at-home sample shipment success, calculated on 103 collected samples, and timing of arrival based on 91 samples received. Percentages are reported as proportion of total collected samples (*) or proportion of samples received (**)

| **VAMS at-home shipment** | | |
| --- | --- | --- |
| Total PwE enrolled | 103 | 100% |
| Success | 91 | *88.35% |
| Arrived in 7 days | 79 | **86.81% |
| Arrived after 7 days | 8 | **8.79% |
| Date not indicated | 4 | **4.40% |
| Failure | 12 | *11.65% |

**Table S3:** Reliability analysis for Aim 1: Bland-Altman and linear regression results comparing VAMS samples collected in ambulatory versus at-home settings with an aliquot quality score ≥3. See relative Figure S2.

|  | Bland-Altman | | | Linear regression | | | | | | | |
| --- | --- | --- | --- | --- | --- | --- | --- | --- | --- | --- | --- |
|  | **Bias** | **95%**  **CI** |  | | **R^2^** | **Intercept** | **95% CI** | **Slope** | **95% CI** | **p value** | |
| CBZ  (n= 14) | -0.70 | -6.09 to 4.69 | y = 4.1201 + 0.3862 x | | 0.46 | 4.120 | 1.863 to 6.387 | 0.386 | 0.131 to 0.625 | | P=0.0074 |
| LCM  (n= 11) | 0.64 | -0.81 to 2.09 | y = 1.0141 + 0.8978 x | | 0.87 | 1.014 | -0.0799 to 2.108 | 0.898 | 0.632 to 1.164 | | P<0.0001 |
| LTG  (n= 37) | -0.23 | -4.10 to 3.64 | y = 0.9668 + 0.8322 x | | 0.96 | 0.967 | 0.3390 to 1.5945 | 0.832 | 0.775 to 0.890 | | P=0.0920 |
| LEV  (n= 23) | -0.39 | -6.70 to 5.92 | y = 2.2435 + 0.7273 x | | 0.81 | 2.244 | 0.322 to 4.165 | 0.727 | 0.567 to 0.888 | | P<0.0001 |

**Table S4:** Reliability analysis for Aim 2: Bland-Altman and linear regression results comparing qDBS and VAMS samples collected in an ambulatory setting (quality score 5).

|  | **Bland-Altman** | |  | **Linear regression** | | | | | | |
| --- | --- | --- | --- | --- | --- | --- | --- | --- | --- | --- |
|  | **Bias** | **95%CI** | **Pearson’s r** |  | **R^2^** | **Intercept** | **95% CI** | **Slope** | **95% CI** | **p value** |
| **CBZ (n= 7)** | -0.09 | -2.351 to 2.177 | 0.71 | y = 1.6019 + 0.7777 x | 0.5 | 1.6019 | -5.343 to 8.547 | 0.7777 | -0.123 to 1.679 | P=0.2327 |
| **LCM (n= 9)** | -0.38 | -1.740 to 0.973 | 0.94 | y = 0.2804 + 0.8610 x | 0.89 | 0.2804 | -1.148 to 1.708 | 0.8610 | 0.585 to 1.138 | P=0.7305 |
| **LTG (n=19)** | 0.14 | -1.845 to 2.188 | 0.93 | y = -0.4167 + 1.1210 x | 0.86 | -0.417 | -1.603 to 0.769 | 1.1210 | 0.885 to 1.357 | P=0.173 |
| **LEV (n=13)** | 1.15 | -2.594 to 4.902 | 0.98 | y = -1.1688 + 1.3266 x | 0.97 | -1.169 | -2.394 to 0.057 | 1.3266 | 1.183 to 1.471 | P=0.3279 |

**Figures**

**Figure S1:** Representative UHPLC-MS/MS Total Ion Chromatograms (TIC) of calibration standards (Level 3) extracted from (A) VAMS and (B) qDBS devices. The peaks show consistent chromatographic behavior and separation for the four antiseizure medications across both sampling platforms. Retention times (RT) are: LEV (1.88 min), LCM (2.82 min), LTG (2.98 min), and CBZ (3.69 min). Optimal chromatographic separation was achieved for all monitored ASMs and their respective internal standards within a 5.5-minute run.

|  | VAMS in ambulatory | VAMS at-home |
| --- | --- | --- |
| Score | 0 | 0 |
| 0 | 0 | 1.0989 |
| 1 | 0 | 1.0989 |
| 2 | 0 | 9.89011 |
| 3 | 0 | 6.59341 |
| 4 | 12.6214 | 7.69231 |
| 5 | 83.4951 | 73.6264 |

**Figure S2:** Histogram showing the distribution of VAMS quality percentages for samples collected in ambulatory settings and at-home, allowing visual comparison of quality across the two collection contexts.

**Figure S3:** Bland-Altman analysis (a) and linear regression (b) for Aim 1, comparing ambulatory and at-home VAMS samples with an aliquot quality score ≥3, assessing reliability between collection settings.

**Figure S4**: Bland-Altman analysis (a) and linear regression (b) for Aim 2, comparing qDBS and VAMS samples collected in an ambulatory setting, assessing reliability between the two microsampling methods.
